# Research on a Community-based Platform for Promoting Health and Physical Fitness in the Elderly Community

**DOI:** 10.1371/journal.pone.0057452

**Published:** 2013-02-27

**Authors:** Tsai-Hsuan Tsai, Alice May-Kuen Wong, Chien-Lung Hsu, Kevin C. Tseng

**Affiliations:** 1 Research Center of Industry Innovation for the Senior Citizens, Chang Gung University, Taoyuan, Taiwan; 2 Department of Physical Medicine and Rehabilitation, Chang Gung Medical Foundation, Taoyuan, Taiwan; 3 Healthy Aging Research Center, Chang Gung University, Taoyuan, Taiwan; Northwestern University, United States of America

## Abstract

This study aims to assess the acceptability of a fitness testing platform (iFit) for installation in an assisted living community with the aim of promoting fitness and slowing the onset of frailty. The iFit platform develops a means of testing Bureau of Health Promotion mandated health assessment items for the elderly (including flexibility tests, grip strength tests, balance tests, and reaction time tests) and integrates wireless remote sensors in a game-like environment to capture and store subject response data, thus providing individuals in elderly care contexts with a greater awareness of their own physical condition. In this study, we specifically evaluated the users’ intention of using the iFit using a technology acceptance model (TAM). A total of 101 elderly subjects (27 males and 74 females) were recruited. A survey was conducted to measure technology acceptance, to verify that the platform could be used as intended to promote fitness among the elderly. Results indicate that perceived usefulness, perceived ease of use and usage attitude positively impact behavioral intention to use the platform. The iFit platform can offer user-friendly solutions for a community-based fitness care and monitoring of elderly subjects. In summary, iFit was determined by three key drivers and discussed as follows: risk factors among the frail elderly, mechanism for slowing the advance frailty, and technology acceptance and support for promoting physical fitness.

## Introduction

Technological and economic developments drive advances in medical technology and the health environment, leading to structural changes in the population. The global population structure has transitioned from an earlier structure characterized by high birth and death rates to low birth and death rates today. If this trend continues, by 2050 Taiwan’s elderly will account for 35% of the total population [1]. Many countries have begun to research and develop medical care and monitoring systems in an attempt to cope with the social trends caused by aging societies. Integrating advances in medical technology with advances in other technological domains allows for the development of assistive devices and systems to enable the elderly to take a more active role in maintaining their health and to live more autonomously [2], [3].

In 2003, National Taiwan University Hospital conducted a national sampling of 2238 individuals over 65 years of age, including 1147 men and 1192 women. Using the frailty test standards proposed by Fried et al. [4], the survey results categorized the respondents as not frail (55.1%), pre-frail (40.0%) and frail (4.9%) [5]. A 2009 survey of 556 individuals aged 65 or above (289 men and 287 women) by the Chinese Medical University found proportions of 22%, 51% and 27% for not frail, pre-frail and frail [6], indicating that the frail and pre-frail proportion of the elderly population has increased over time. Properly intervening prior to the onset of frailty requires an understanding of the relevant definitions and contexts of frailty. Frailty is not a disease, although testing for frailty can give insight into the patient’s physical condition. However, frailty is a part of the aging process and is an unavoidable physiological change [7]. Frailty causes the gradual degeneration of physiological functions in elderly people, including reduced endocrine and metabolic function, gradual muscle atrophy, reduced appetite, poor memory skills and reduced mobility [8], [9]. In addition, the study shows that the onset of frailty in the average person begins at age 45, most noticeably with a decline of bodily functions [10]. With increasing age, physical frailty is inevitable, but the onset of frailty can be prevented or delayed [11]. Thus, from the perspective of prevention, actively physical training can preserve bodily functions, while preventing and mitigating their decline, thus reducing the incidence of disease [12].

Exercise is an integral part of frailty prevention [13]. For example, cardiovascular endurance training can help prevent cardiovascular disease, heart disease, arrhythmia, and hypertension. Strength training also helps avoid the aging of muscle tissue, prevents osteoporosis and muscle wastage, and increases muscle tone and joint flexibility [14], [15]. Psychologically, regular exercise can improve self-perception, alleviate depression, and mitigate self-control issues. However, prior to providing assistance, the patient needs to be evaluated in terms of previous medical history and drug use to formulate the most appropriate exercise plan. For example, the American Sports Medicine Institute proposed four variables for exercise including duration, frequency, type and intensity. The recommended frequency is every other day, at least three times per week or multiple times during the day. Before increasing exercise intensity, they recommend first increasing exercise duration, and exercise intensity must match the conditions of low risk or high risk chronic illness in the elderly. In addition, the elderly frequently interrupt their regular exercise for various reasons, such as concerns over injury, and thus have difficulty developing sustainable exercise habits.

However, due to variation in the manifestation of frailty between patients, no unified set of test items or standards have been developed [16]. Generally speaking, tests for frailty can be divided into two kinds: physiological and psychological. The physiological tests assess whether the patient is able to maintain activity of daily living (ADL) [17]. The psychological tests primarily include cognitive and emotional assessments [18]. At present, the most-widely accepted frailty test was developed by Fried et al. in 2001 [4]. However, some scholars have criticized this method as overly simple and have proposed alternative methods. For example, Campbell and Buchner have suggested that frailty requires additional consideration of musculoskeletal function, aerobic capacity, cognitive function and nutritional status [19]. Rockwood et al. divided aging into seven degrees, requiring observation of as many as 70 items [20]. Furthermore, differences in ethnic, cultural and geographic contexts results in differences among similar test items, item content and assessment values. The comprehensive geriatric assessment (CGA) provided by the American Geriatrics Society divides health assessments for the elderly into two major steps [21]. First, various specialists from different areas diagnose and describe the patient’s condition. Next, geriatrics specialists prioritize these conditions for treatment. The primary assessment items of the frailty index of comprehensive geriatric assessment (FI-CGA) include cognitive, emotional communication skills, mobility, balance, bowel function, bladder function, daily life skills, nutrition and social resources [22]. Patient regression in these areas is classified as mild, moderate or severe. The assessment of social resources is mainly divided into environmental and medical factors. The environmental factors evaluate the safety of the patient’s home environment, while the medical factors primarily involve a detailed comprehensive history of the patient’s physical examinations [23]. Therefore, to ensure that the test items and assessment data are well-suited to people in Taiwan, this study uses health and fitness standards issued by the ROC Bureau of Health Promotion [24].

To this end, this study aims to assess the acceptability of a fitness testing platform (iFit) for installation in a community with the aim of promoting fitness and slowing the onset of frailty. Technology acceptance among elderly people of this type of technology will be assessed, especially in terms of ease of use and the suitability of game play. In consideration of the potential reluctance of elderly people to use the proposed fitness testing platform, this study used the technology acceptance model (TAM) [25] to assess the users’ degree of technology acceptance. We hypothesized that the proposed platform can offer user-friendly solutions for a community-based fitness care and monitoring of elderly subjects. In addition, the proposed platform was expected to promote health and physical fitness in the elderly community.

## Methods

### Subjects

**Figure 1:**
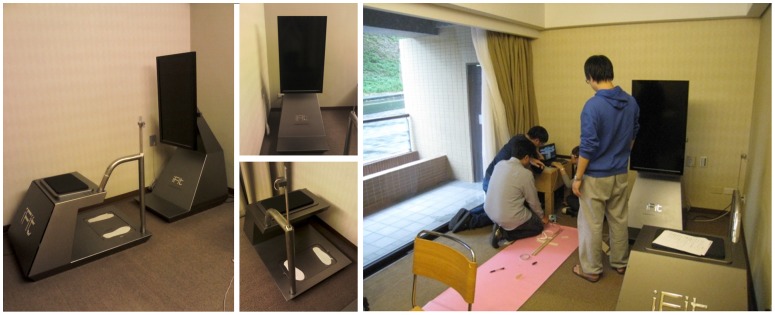
iFit fitness testing platform.

The proposed iFit fitness testing platform was installed for clinical testing in the Chang Gung Health and Culture Village as shown in Figure 1. We recruited 101 subjects, aged 60 or above (N = 530, 30 September 2012), with a male/female ratio of 26.7% to 73.3% from Chang Gung Health and Culture Village. Subjects with a history of psychological problems, mental and motor dysfunctions, or cognitive deficits were excluded from this study. Others were excluded on a case by case basis on account primarily of severe cardiovascular disease, cognitive impairment or medical conditions rendering them unable to participate or use the system. We obtained ethics approval for our study from the Institutional Review Board (the “IRB”) of Chang Gung Medical Foundation. All relevant ethical safeguards have been met in relation to patient or subject protection. After we meticulously explained the purposes of this study to the subjects, all subjects signed informed consent forms before participating. In addition, the subjects of the photographs in this manuscript have given written informed consent to publication of their photographs.

### Procedures

Subjects were first introduced to the platform and guided through its usage. Detailed explanations were given regarding traditional testing items including grip strength, flexibility, balance and reaction, along with the corresponding iFit games. iFit testing focused primarily on four test items:

- Flexibility test: the subject sits on the platform and stretches forward. Subjects not in the habit of exercising were prompted to warm up prior to testing to avoid possible injury during stretching.
- Grip strength test: the subject tightly grips an object, rests for two minutes and then repeats, taking the average of the two instances.
- Balance test: the subject stands on one foot with his eyes closed, with the platform recording the duration in seconds. The subject then rests for two minutes and repeats, taking the average of the two instances.
- Reaction time test: the subject sits on the platform, recording the time and required to catch the object.

**Figure 2:**
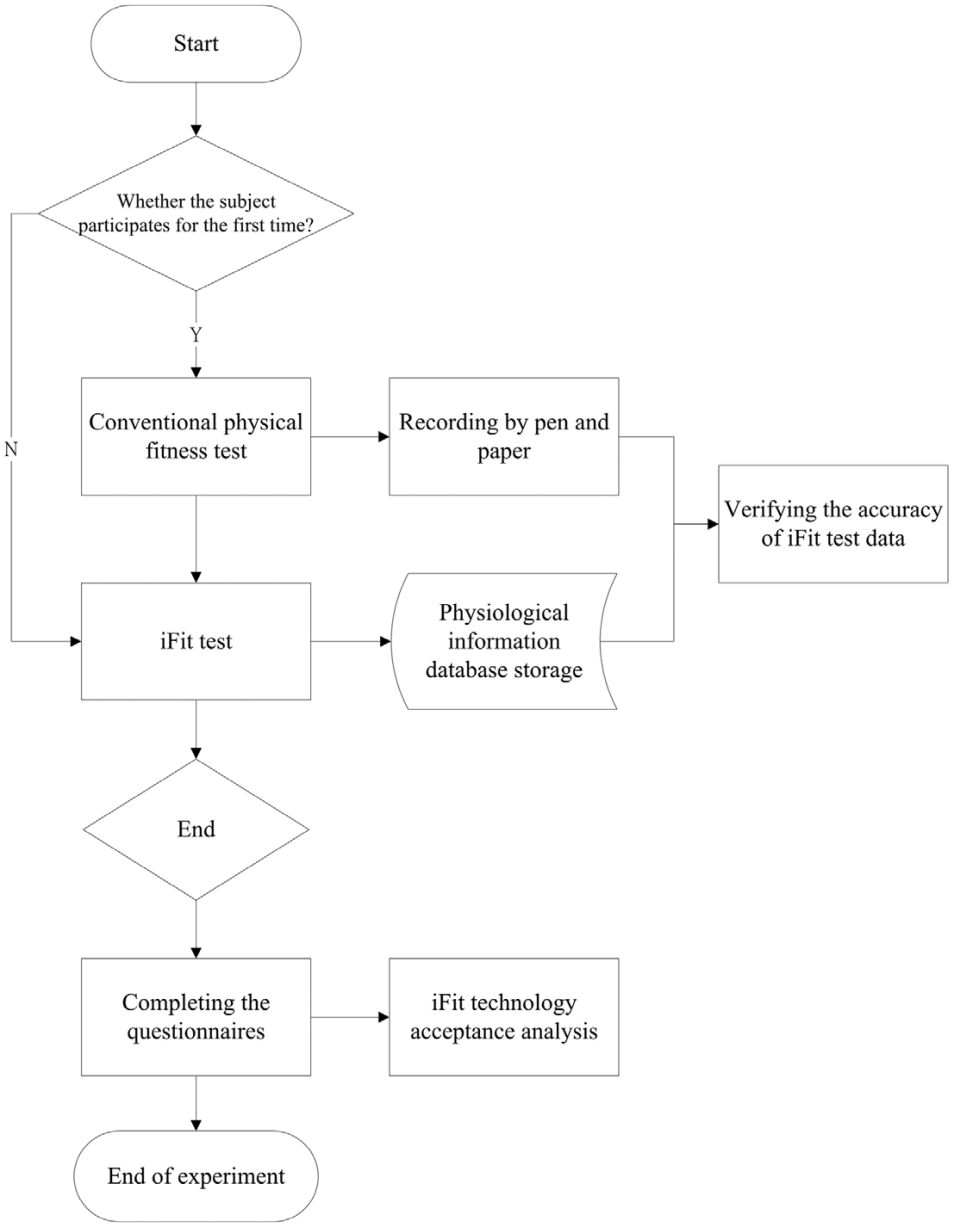
The experimental procedure.

**Figure 3:**
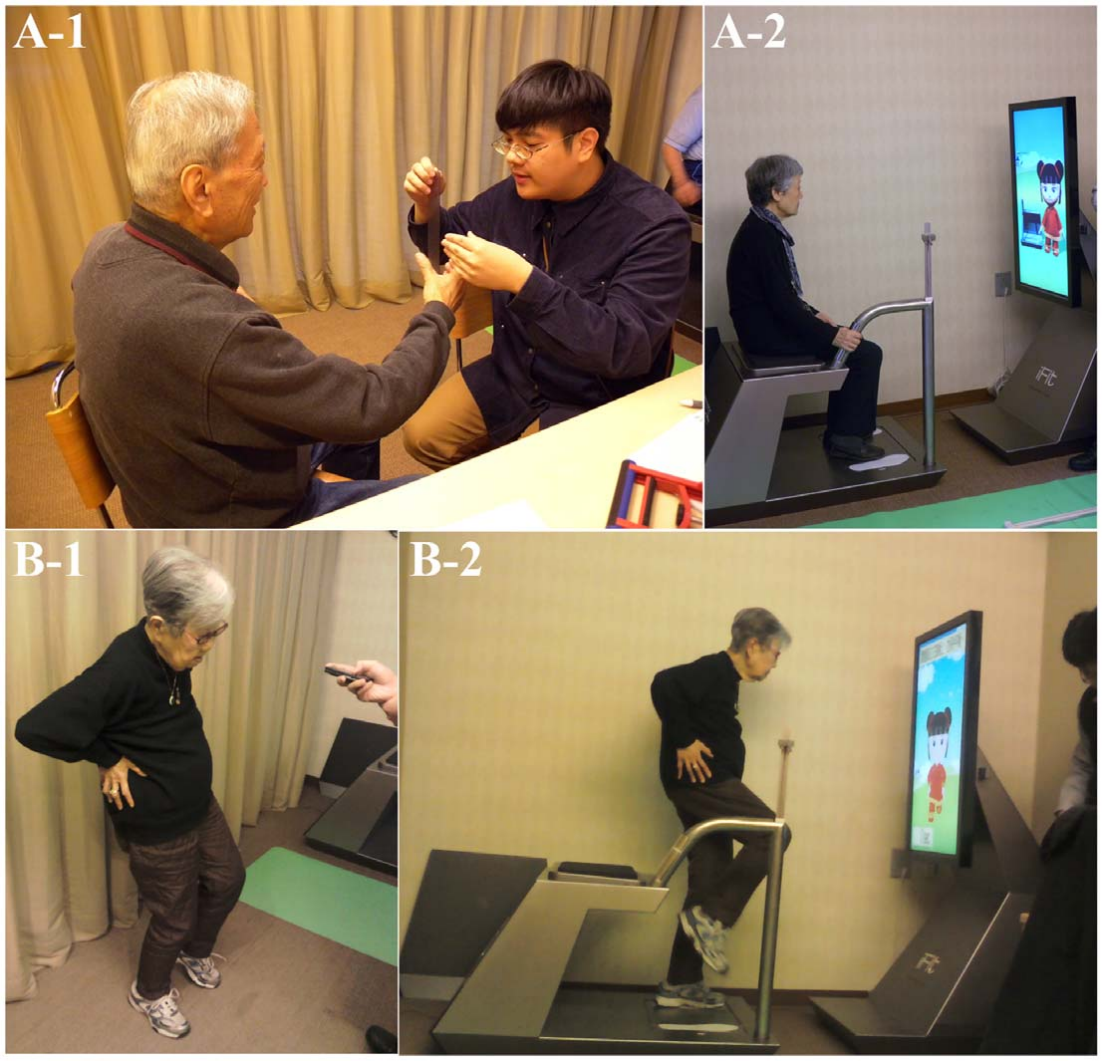
The experimental procedures for the traditional and iFit tests. (A-1) The conventional reaction-time measurement. (A-2) The iFit reaction-time measurement. (B-1) The conventional balance measurement. (B-2) The iFit balance measurement.

Figure 2 illustrates the experimental procedure. The researchers first introduced the platform and showed a video depicting the experimental process. Researchers also determined whether it was the first time the subjects had participated in fitness testing, and then guided first time subjects in complete a traditional fitness test, the data from which were recorded. Next, the subjects completed the iFit test. In this process, the research team members did not need to record data and could focus primarily on assisting the subjects in completing the test process. Finally, the subjects were asked to complete a questionnaire. Figure 3 shows the formal experimental procedures for the traditional and iFit tests. Subject suggestions and feedback on the process, obtained either through the questionnaires or through video recorded interviews, were collected for future improvement of the iFit platform.

### Hypotheses and Validation

**Figure 4:**
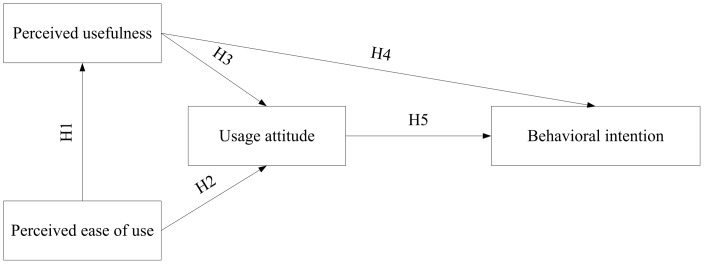
The research model.

To test the acceptance of the iFit by elderly users, this study installed the platform in the Chang Gung Health and Culture Village. A TAM-based questionnaire was used to survey elderly user acceptance of and intent to use the iFit, and to verify factors supporting intent to use. The model structure is shown in Figure 4.

This research included three independent variables (perceived ease of use, perceived usefulness and usage attitude) and studied the influence of these variables on behavioral intention. The following hypotheses are proposed:

- H1 “Perceived ease of use” of the iFit platform has a positive and significant impact on “perceived usefulness”.
- H2 “Perceived ease of use” of the iFit platform has a positive and significant impact on “usage attitude”.
- H3 “Perceived usefulness” of the iFit platform has a positive and significant impact on “usage attitude”.
- H4 “Perceived usefulness” of the iFit platform has a positive and significant impact on “behavioral intention”.
- H5 “Usage attitude” for the iFit platform has a positive and significant impact on “behavioral intention”.

These hypotheses reference previous research findings regarding technology acceptance questionnaires, primarily in discussing the dimensions of perceived ease of use, perceived usefulness and usage attitude to determine whether these variables influence the user’s intention to use the iFit. The definitions of each variable and questionnaire item aid the understanding of the user’s intention and behavior regarding the iFit. First, perceived usefulness suggests that the technology will assist the user increase the effectiveness of his work. Providing appropriate information encourages the user to believe that using the platform can help him increase his understanding of the physiological data. Increased perceived usefulness correlated with a positive usage attitude. This study used a 5-point Likert scale (1 = strongly agree ∼ 5 = strongly disagree). Second, perceived ease of use indicates the user believes that using the system does not require significant effort. When the user perceives the system as being easier to use, it increases the positive attitude dimension which, in this study refers to the user’s degree to which the user perceives the platform as being easy to use. Third, behavioral attitude refers to a user’s identification of a given behavior type as being good or bad, or positive or negative. The user’s attitude towards a given type of behavior is influenced by the behavioral beliefs and evaluation of results when executing that behavior which, in this research, refers to the user’s positive or negative evaluation of the platform. Fourth, behavioral intention refers to the strength of the user’s willingness to use engage in a particular behavior. Predicting whether an individual will engage in a particular behavior requires understanding his behavioral intention. A very strong relationship exists between behavioral intention and actual behavior [26], [27]. Thus the measurement of actual behavior can serve as a proxy for behavioral intention, referred to as the intentional mode. In this study, this dimension refers to the willingness of the user to use the platform.

### Statistical Analysis

During the testing period, questionnaire responses were processed continuously. Questionnaires were first reviewed to ensure completeness and validity, thus preventing data input errors. Each completed questionnaire was given a serial number and then input into an SPSS data file. When the valid questionnaires were completely inputted, they were reviewed a second time to correct input errors. Of the 101 elderly respondents, 26.7% were male and 73.3% were female, and with an average age of 79 (see Table 1). Of the total respondents, 52.5% had previously undergone fitness tests, while 30.7% had previously played interactive games. While many of the elderly respondents had a certain degree of experience with fitness tests, they were less familiar with the use of interactive games for testing or training.

**Table 1:** Statistics of age and degree of education.

	Age
Mean	79.6
n	101
S.D.	7.5
Min.	60.0
Max.	93.0

## Results

To verify that each questionnaire item was statistically significant, this study first conducted descriptive statistics for each question. The results are presented in Table 2, in which the descriptive statistics section shows each question scoring on average between 3.9∼4.2. This indicates that TAM in this study tends towards accepting the use of the fitness testing platform. However, the reliability and validity of the questions is validated by confirmatory factor analysis (CFA), and structural equation modeling verifies the influence and paths between the dimensions.

**Table 2:** Descriptive statistics of TAM items (n = 101).

	Min.	Max.	Mean	S.D.	% ofScore 5
Perceived usefulness 1	2.0	5.0	3.9	.8	18.8
Perceived usefulness 2	3.0	5.0	4.0	.6	17.8
Perceived usefulness 3	3.0	5.0	4.1	.5	16.8
Perceived usefulness 4	3.0	5.0	4.2	.5	25.7
Perceived usefulness 5	3.0	5.0	4.1	.5	21.8
Perceived ease of use 1	2.0	5.0	4.1	.6	21.8
Perceived ease of use 2	2.0	5.0	4.1	.6	25.7
Perceived ease of use 3	2.0	5.0	4.1	.6	22.8
Perceived ease of use 4	2.0	5.0	4.2	.6	25.7
Perceived ease of use 5	2.0	5.0	4.2	.6	26.7
Perceived ease of use 6	3.0	5.0	4.2	.6	29.7
Behavioral intention 1	3.0	5.0	4.2	.5	21.8
Behavioral intention 2	2.0	5.0	4.1	.6	25.7
Behavioral intention 3	3.0	5.0	4.1	.6	23.8
Behavioral intention 4	2.0	5.0	4.1	.6	24.8
Usage attitude 1	3.0	5.0	4.2	.6	28.7
Usage attitude 2	2.0	5.0	4.1	.7	28.7
Usage attitude 3	2.0	5.0	4.1	.7	24.8
Usage attitude 4	2.0	5.0	4.1	.6	24.8
Usage attitude 5	2.0	5.0	4.0	.7	24.8

Prior to factor analysis, we first obtained the Kaiser-Meyer-Olkin measure of sampling adequacy (KMO) and Bartlett’s sphericity test to determine whether the items were suited to factor analysis. KMO values are between 0 and 1, with values closer to 1 indicating a higher degree of relatedness and thus greater suitability for factor analysis [28]. In Bartlett’s spherical test, if the variable’s correlation coefficient is higher, the obtained χ2 is greater, and P<0.05 indicating greater suitability for factor analysis [29]. Analysis results are presented in Table 3, with a KMO value of 0.929 and a Bartlett spherical test P<0.001, thus meeting the abovementioned criteria. Thus, the questionnaire designed using the TAM model design is suitable for further factor analysis.

**Table 3:** KMO and Bartlett’s sphericity test.

Kiser-Meyer-Olkin measure of sampling adequacy		.929
Bartlett’s sphericity test	Approximately Chi-Square	1516.571
	Degree of freedom	190
	Significant	.000***

*** : p<.001.

Next we calculated the factors using the Varimax method, with the factor loadings shown in Table 4. Aside from behavior attitude, which produces a value of 0.43, the factor loadings of the other variables are all above 0.5. Generally, when the number of samples exceeds 100, a factor loading value of greater than 0.5 is considered to be acceptable for convergent validity and discriminant validity. Thus, we omit the attitude question.

**Table 4:** Rotated component matrix.

Factors	1	2	3	4
Perceived usefulness 1	.124	.297	.406	.585
Perceived usefulness 2	.113	.109	.252	.809
Perceived usefulness 3	.401	.373	.139	.623
Perceived usefulness 4	.423	.407	.192	.601
Perceived usefulness 5	.123	.307	.234	.756
Perceived ease of use 1	.762	.059	.324	.099
Perceived ease of use 2	.747	.214	.127	.096
Perceived ease of use 3	.850	.090	.224	.186
Perceived ease of use 4	.647	.472	.175	.134
Perceived ease of use 5	.505	.620	.165	.213
Perceived ease of use 6	.729	.351	.223	.276
Behavioral intention 1	.251	.800	.153	.216
Behavioral intention 2	.178	.763	.267	.280
Behavioral intention 3	.138	.709	.365	.308
Behavioral intention 4	.181	.527	.589	.259
Usage attitude 1	.289	.554	.431	.302
Usage attitude 2	.325	.158	.808	.223
Usage attitude 3	.277	.149	.775	.237
Usage attitude 4	.109	.434	.710	.242
Usage attitude 5	.285	.235	.796	.217

According to the verified factor analysis results, the TAM model’s path structure analysis are presented in Table 5. The analysis results for each indicator show a chi-square value of 226.110, df = 146, p<0.0001, a chi-square degree of freedom of 1.549, which is less than 3 [30]. The CFI (Bentler’s fit index) value is 0.941, which is greater than 0.9 [31]. The incremental fit index (IFI) value is.942, which is greater than 0.9 [32]. The root mean square residual (RMR) value is 0.024, smaller than 0.025 [30]. The root mean square error of approximation (RMSEA) value is 0.074, smaller than 0.08 [33]. Thus all indicators are in compliance with the general standard requirements, indicating a good fit for the structural model.

**Table 5:** TAM model’s path structure analysis.

Fit index	χ^2^	*df*	χ^2^/*df*	CFI	IFI	RMR	RMSEA
	226.110	146	1.549	0.941	.942	0.024	0.074
Recommended value			<3	>0.9	>0.9	<0.025	<0.08

**Figure 5:**
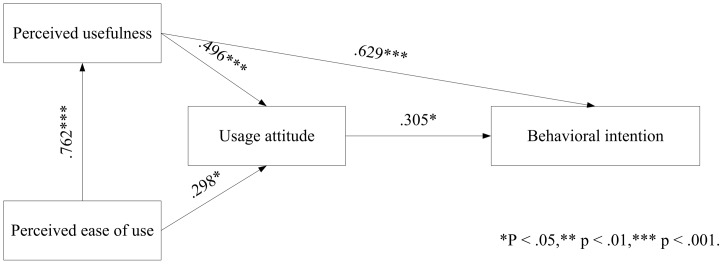
Assumptions verification figure.

Verification of proposed research’s reliability is followed by hypothesis testing analysis. This study used confirmatory factor analysis [34] through structural equation modeling to analyze the proposed hypotheses [35], through structural equation evaluation of the correlation coefficient and significant path for each factor. This five hypotheses proposed here had a T-value reliability standard of 95%. The statistical analysis and results shown in Table 6 and Figure 5 show the usage intention of elderly people for the fitness test platform is positive and significant. Results are as follows:

**Table 6:** Hypotheses validation.

Hypotheses	Description	Standardizedregression weights	T-values	Result
**H1**	“Perceived ease of use” of the iFit platform has a positive andsignificant impact on “perceived usefulness”	0.762	7.40***	Support
**H2**	“Perceived ease of use” of the iFit platform has a positive andsignificant impact on “usage attitude”.	0.298	2.105*	Support
**H3**	“Perceived usefulness” of the iFit platform has a positive andsignificant impact on “usage attitude”.	0.496	3.304***	Support
**H4**	“Perceived usefulness” of the iFit platform has a positive andsignificant impact on “behavioral intention”.	0.629	4.824***	Support
**H5**	“Usage attitude” for the iFit platform has a positive andsignificant impact on “behavioral intention”.	0.305	2.700*	Support

* p<.05,

*** p<.001.

- H1 holds that “perceived ease of use” of the iFit platform has a positive and significant impact on “perceived usefulness”. The results support H1, indicating that the “perceived ease of use” of the proposed platform has a positive and significant impact on the iFit’s “perceived usefulness”.
- H2 holds that “perceived ease of use” of the iFit platform has a positive and significant impact on “usage attitude”. The results support H2, indicating that the “perceived ease of use has a positive and significant impact on the “usage attitude” of iFit users.
- H3 holds that “perceived usefulness” of the iFit platform has a positive and significant impact on “usage attitude”. The results support H3, indicating that the “perceived usefulness” of the proposed platform has a positive and significant impact on the “usage attitude” of iFit users.
- H4 holds that “perceived usefulness” of the iFit platform has a positive and significant impact on “behavioral intention”. The results support H4, indicating that the “perceived usefulness” of the proposed platform has a positive and significant impact on the “behavioral intention” of iFit users.
- H5 holds that “usage attitude” for the iFit platform has a positive and significant impact on “behavioral intention”. The results support H5, indicating that the “usage attitude” of users of the proposed platform has a positive and significant impact on the “behavioral intention” of iFit users.

## Discussion

In recent years, combining games with sports has become an important direction in game development, with new games and game platforms integrating various new sensing technologies including infrared imaging, gyroscopes and tools to capture the user’s physiological signals for use in virtual reality applications. Today, games are no longer played sitting down, but rather encourage people to interact with the game through a range of physical movement, increasing the physical activity required from the player. These functions create opportunities for physical exercise and social interaction, and have largely replaced the repetitive motion characteristic of traditional games. For example, Nike and Nintendo jointly developed shoes with a built-in balance sensor for use in virtual reality games for the Wii game platform to promote movement and interaction [36]. Sony uses sensors combined with the traditional handlebar controller to develop somatosensory games. Despite the game interface being in 3D, the controller and game play are similar to that of the Wii [37]. Microsoft’s Kinect uses image recognition and 3D depth sensing to capture the user’s position and body movements, allowing the user to control the game with gestures and voice commands without the need to wear or hold sensors [38]. Taiwan’s Eco-City project created a virtual reality bicycle application which provides elderly users with cardiopulmonary, strength, endurance, and spatial awareness training to slow the deterioration of spatial awareness as people age [39]. Based on the abovementioned studies, the majority of current interactive somatosensory games are aimed at the general user. While the point of the game design is to increase movement, improper use can result in muscle strain. Hirpara and Abouazza pointed out that Wii somatosensory games can easily lead to muscle fatigue, strained eyesight, damage to nearby objects, accidents and addictive behavior [40]. Sparks et al. also pointed out that Wii somatosensory games could easily lead to 14 different types of injuries, including hand lacerations, which accounted for 44% of total injuries [41]. The abovementioned games are all sports-oriented, though few explain the proper motion intensity or duration in the course of the game, which is a significant concern for use among elderly people who are already weaker than the average user. Thus, this research considers the iFit for use as a community-based fitness assessment platform, with virtual reality games based on Bureau of Health Promotion standards and designed to suit the needs and capabilities of elderly users.

Therefore, this study proposed a technology acceptance model, applied to the behavioral intention of elderly people to use a fitness testing platform. Research results confirm that perceived usefulness, perceived ease of use and usage attitude positively impact behavioral intention to use the platform. In addition, user intention to use the platform a second time rises with perceived usefulness, and perceived ease of use also has an indirect impact on the behavioral intention degree of acceptance through usage attitude: increasing the ease of use positively impacts the usage attitude and intention among elderly users. The study results also suggest that usage attitude has a positive impact on usage intention.

The findings shown in this paper are also supported by Goodman et al. [27], Theng et al. [42], Chen et al. [43] and Holzinger et al. [44]. In addition, Walsh and Callan [26] determined the influential factors, i.e. users’ perception, experiences, and familiarity with technology, that affect the attitude of users. Theng et al. [42] further divided over the attitude into emotional response, sociability, and satisfaction. In terms of senior fitness and exercise, the improvement or maintenance of physical fitness is required to accomplish continuous and periodic trainings. Older people’s perception of ease of use and usefulness is announced in this study and, most significantly, the extent to which older users believe using the technology will benefit them. In summary, iFit was determined by three key drivers and discussed as follows: risk factors among the frail elderly, mechanism for slowing the advance frailty, and technology acceptance and support for promoting physical fitness.

- Risk factors among the frail elderly: iFit enables the elderly to measure fitness factors including grip strength, flexibility, balance, and reaction time. Those factors are indications of essential health status of people. In long-term recording, the elderly, caregivers, and doctors can realize whether the elderly have improved their physical fitness ability.
- Mechanism for slowing the advance of frailty: It provides a literature-based investigation of mitigation mechanisms for risk factors for the elderly and, through analysis of fitness testing data, provides recommendations for the further improvement of game-based fitness promotion, thus promoting the development of customized exercise plans based on analysis of physical frailty, so that the fitness testing platform can again be integrated into the health data community for the elderly.
- Technology acceptance and support for promoting physical fitness: Through introducing a community-based fitness testing platform to test for four elderly fitness functions, this study uses a technology acceptance model to verify the usage intention of elderly users for the platform. Results indicate that the platform introduction is successful in that the elderly users indicate a high perceived usefulness and perceived ease of use for the platform. These two dimensions are significantly and positively correlated to user attitude, while perceived usefulness and usage attitude are significantly and positively correlated to usage intention, providing a good indication of continued use.
